# Choice, control and person-centredness in day centres for older people

**DOI:** 10.1177/1468017320952255

**Published:** 2020-08-30

**Authors:** Katharine Orellana, Jill Manthorpe, Anthea Tinker

**Affiliations:** King’s College London, England

**Keywords:** Social work, older people, personalisation, community care, qualitative research

## Abstract

**Background:**

Day centres are a substantial element of community-based support for older people in many countries. However, assumptions that they are an outdated or costly service model have resulted in many centre closures in England. The perspectives of 42 people attending, providing, making referrals to or purchasing places at four diverse day centres for older people were collected in interviews. Using these data, we explore day centres’ relevance to social workers’ efforts to promote person-centred support for older people enabling them to maintain or improve their well-being. These are explored from the perspectives of choice, control and person-centredness and local authority responsibilities for shaping the care market under the Care Act 2014.

**Findings:**

Attenders highly valued centres’ congregate nature and the continuity they offered which contributed to the development of person-centred relationships. Attenders exercised choice in attending day centres. Social work staff were more positive about day centres’ relevance to personalisation than those responsible for making decisions about the shape of local care services.

**Applications:**

With social isolation recognised as a serious risk of old age, the value of togetherness in group environments may need highlighting. Enactment of personalisation policies need not necessarily lead to individualisation; day centres may be community-based assets for some. Those shaping the care market may be encouraged to acknowledge wider outcomes, and frontline social workers may benefit from hearing positive experiences that may help in the development of effective care plans for older people who would like to benefit from day centre participation.

## Introduction

Day centres for older people are community building-based services that provide care and/or health-related services and activities specifically for older people who are disabled or in need of support. Attendance can be for a whole or part of a day and cover any number of days. Centres offer various activities that may be considered ‘preventive’ of decline or ill-being. They have been an integral part of English social care services in local government and the voluntary sector since the National Assistance Act 1948 (HM Government, 1948), and are widely found internationally (see Orellana et al., 2020b). While some criticisms and negative depictions of day centres relate to their congregate nature (see Section ‘Background’), our recent scoping review of the international evidence (Orellana et al., 2020b) identified several indications that their congregate nature may contribute to positive outcomes. They may be part of community-located assets supporting many older people’s wishes to age in place.

The English government adopted personalisation as a policy centred on the principles of individual choice and control (HM Goverment, 2007) that was to be accomplished via person-centred support planning and individual funding mechanisms. The implementation of personalisation took place alongside substantial social care funding contractions in England (Ismail et al., 2014). One consequence of funding pressures has been a substantial reduction in the numbers of older people entitled to publicly funded social care (Ismail et al., 2014) and increases in the cost of social care provision for self-funders and for people using publicly funded social care services alike. Another consequence has been cuts in the commissioning of services such as day care from non-governmental organisations and community groups. Meanwhile, while social care policy is to support people to age in place (HM Government, 2014), increasing numbers of older people are living alone (Kempton & Tomlin, 2014), with those aged 85 or older at greatest risk of social exclusion (Key & Culliney, 2018).

The Care Act 2014 requires English local authorities to shape the local care market (Needham et al., 2017). Evidence-based commissioning of care services is encouraged (Local Government Association, 2015), yet some services, such as day centres, have an under-developed evidence base (Allen & Miller, 2013). Given this context, the views of people involved with day centres may help social workers contribute to the market shaping role of their employers as well, enrich their practice in planning support with older people and empowering them to make choices.

This article explores day centres’ relevance in the Care Act 2014 context from the perspectives of the key tenets of choice, control and person-centredness. Focusing on the perspectives of centre attenders and local authority social work staff and commissioners (responsible for market shaping), we draw on findings of a three-year case study of four English ‘generalist’ day centres for older people, those not offering specialist care to a specific group such as people living with dementia or stroke survivors. In the ‘Background’ Section, we synthesise criticisms of day centres, the contemporary (early 2020 and pre-COVID-19 context) policy and funding context and its impact on day centres, the research context and outline our study. The ‘Findings’ Section first sets out social workers’ and commissioners’ views concerning the relevance of day centres to the policy of personalisation. It then explores how person-centredness is enacted at day centres and in their access. Finally, it reports attenders’ favourite and least favourite aspects of their day centres, most of which relate to their group environment and the continuity they afford, highlighting the positives and drawbacks of these. After discussing day centres’ congregate nature, the benefits of the continuity, and choice and control experienced, we draw conclusions for practice and policy that may be relevant to other jurisdictions and make suggestions for future research.

## Background

Day centres are reputedly an ‘old fashioned’ and ‘institutionalised form of service’ (Lloyd et al., 2014, p. 34). Just as residential institutions were stigmatised as undermining people’s self-identity (Goffman, 1961) and for operating structures that created and reinforce dependency (Townsend, 1981), day centres have sometimes been characterised by similar organisational structures and labelled as institutions, albeit ‘partial’ institutions as they only operate during the day (Salari, 2002; Townsend, 1981). They have been described as perpetuating social isolation in a different setting ‘out of normal community life’ (Tester, 1989, p. 168). These negative depictions of day centres endure; for example, they have been described as ‘desolate places’ in which people are unlikely to make friends (Cottam, 2009), and places which reinforce dependency and which provide care to passive recipients (Needham, 2014). Internationally, day centres appear to be, mainly, more positively viewed. For example, in Singapore, they are considered key enablers of ageing in place (Liu et al., 2015); in the Czech Republic, they are a place in which to meet people and engage in activities (Marhankova, 2014); in Norway, they support the prevention of loneliness and isolation (Boen et al., 2010; Lund & Englesrud, 2008); and in Israel, they are part of a package of community services covered by long-term care insurance as services which support the well-being of socially isolated, frail people who are ageing in place (Iecovich & Biderman, 2013; Ron, 2007).

Elsewhere, we have set out the changes resulting from the introduction of a social care ‘market’, a policy of ‘personalisation’ and public funding cuts on generalist day centres for older people (Orellana et al., 2020b). Such moves in England were further confirmed by the Care Act 2014 (HM Government, 2014). This Act requires English local authorities to enable people to access the support they need to promote well-being and help prevent or delay deterioration (financially means-tested), and to shape a market that delivers a wide range of care and support options. It continues policy themes such as promotion of well-being, prevention of decline, strengths-based approaches and stimulation of voluntary or community support for older people and carers, and enabling choice about remaining at home while growing older, ‘ageing in place’.

Under the Care Act 2014, those eligible for publicly funded social care are entitled to exercise choice and control over their care and support, with an intended outcome that this will better meet their needs and preferences, and to sustain their independence and social participation. Personalised care and support would be enabled by person-centred or strengths-based assessment and support planning, and individualisation of finances. Such developments are not confined to England but are internationally observed, particularly in relation to the individual ‘cash for care’ model whereby an eligible person chooses to take Direct Payments and arranges their own care and support (FitzGerald Murphy & Kelly, 2019).

Many authors, in UK social work and wider, have highlighted how the local authorities’ pressing needs to make financial savings may have overshadowed the individual choices that the policy of personalisation and the Care Act 2014 were intended to enable (e.g. Barnes, 2011; Fine, 2013; Needham, 2014; Needham & Glasby, 2015). At one extreme, some have argued that group services are inappropriate, in a personalised environment, and are unresponsive to individual needs and preferences (Barnes, 2011). However, Stevens et al. (2011) argued that even presenting choice in social care as ‘individual’ conflicts with its public nature. How one person’s choice may affect another’s options can be exemplified:

For example, if 30 users of a local authority day centre decide not to use that service it may well become unprofitable and have to close, denying the choice of another 30 users that the service should remain unchanged. (Lymbery & Postle, 2015, p. 83).

Between 2010–2011 and 2013–2014, English day centre places dropped by 66.9% (178,700 to 59,125) (Age UK, 2015). These numbers refer to older people using local authority-provided or commissioned services; the numbers of people self-funding their centre attendance are unknown. In 2018, figures collected from Freedom of Information requests to local authorities revealed public funding cuts, in over 40 responding areas, of up to 55% to day centres in the past five years, with an average funding reduction of 30% (Green, 2018).

Widespread closures of day centres are purported to be, in part, a justifiable response to personalisation which, some believe, render day centres an outdated service model (see Needham, 2014) and one which is no longer a local authority core service. However, commissioning decisions are not always based on evidence or service user feedback (Miller et al., 2014) despite market shaping requiring local strategic planning to be evidence-based, and to include market and local needs analyses.

Of course, day centres are not the only community-based option. However, services or support accessed through local authorities must meet any needs identified in an assessment and be approved by the local authority as the ‘care package’. Employing a Personal Assistant or directly employed care worker is another option for support with user-defined activity. Alternative socialising options include community centres offering programmes or drop-in activities, lunch clubs, University of the Third Age groups, and social or interest groups operated by faith groups, charities, or membership organisations. Given public sector financial constraints, most of these are not supported financially by local authorities. Furthermore, they tend not to offer personal care, lack disability accessible facilities and do not provide transport; some attenders in this present study had withdrawn from such activity and most had become socially isolated because of declining health and mobility (Orellana et al., 2020a).

### Research context of the study

National data on day centres are difficult to obtain in England, as day centres are not regulated. This contrasts to the United States (US), for example, where day centres are integrated within the health and care system, operational models are clear cut and where a professional membership association (National Adult Day Services Association) undertakes large-scale surveys (Orellana et al., 2020b). The most recent major study of day centres for older people in England and Wales was over 30 years ago and aimed to facilitate day care policy development at a time when such centres were mostly local authority run or funded (Tester, 1989). However, under the NHS and Community Care Act 1990, most English local authority services were required to transfer to the independent sector and the social work role changed to care management and arrangement of packages of care. The overarching aim of supporting well-being promoted by the Care Act 2014 sees the social work role as needing to shift focus by moving to strengths-based approaches and maximising local assets (Whittington, 2016). The present article explores the potential for day services to be part of such developments and contributes to the emerging literature on the early implementation of the Care Act 2014.

## Methodology

In order to gain an insight into day centres in their contemporary context, the study reported here took an embedded multiple-case study approach (Cresswell, 2013; Stake, 1995; Yin, 2014). Between September 2015 and December 2016, the first author undertook weekly visits to four day centres for 14 weeks each, and interviewed 13 local authority social work or other staff responsible for service commissioning or making referrals, 23 older attenders, 10 family carers and 23 day centre managers, frontline volunteers and staff. To increase heterogeneity, centres were purposively recruited to reflect organisational and area differences spanning local authority, housing association (social renting) and two voluntary or not-for-profit sector centres, with attendance ranging from 6 to 28 people on the visit day, accepting open and/or local authority referrals, and operating for one, two and five days, two in highly urban areas, one in a small town and one in a rural area, with a range of deprivation levels.

Ethical approval was obtained from the Health Research Authority’s Social Care Research Ethics Committee, and local authority Research Governance approvals were granted. All eligible to participate were given a study Information Sheet, invited to participate and given the opportunity to ask questions. Each participant gave informed consent in writing before the interview. Inclusion criteria were attendance on the researcher’s ‘visit’ day, with the ability to understand hypothetical situations and give informed consent. Just over half of all attenders present on visit days (n = 68) met these criteria (n = 37, 54%). Two-thirds of those eligible (62%, n = 23) and one-third (34%) of all attenders participated. Local authority social services professionals (n = 13) from the centre localities were recruited using snowballing techniques. Gatekeeping was evident among some social workers, with some managers declining to circulate study information to their staff which led to no social work participants in one area.

Face-to-face interviews were undertaken with field notes made afterwards. Interview questions for local authority participants appear in Table 1. Attender interviews (see Orellana et al., 2020a for interview schedule) explored outcomes of day centre attendance, including identifying any outcomes participants would not have experienced if not attending their day centres, feelings about and experiences of attending. To establish the most valued outcomes or experiences, attenders were asked to describe the two things they liked best about going to a day centre. They were also asked about the two things they liked least and whether there was anything they would change. Interviews also covered finding out about and accessing centres, preconceptions of them, first experiences, views on the activities, co-attenders and people providing the service, dealing with problems arising, involvement in operation, payment, value for money and whether they planned to continue attending and would recommend attending to others in a similar situation. Interviews took place in attenders’ homes, private spaces at day centres or local authority offices. Interviews were recorded and transcribed. Using pseudonymised data uploaded to NVIVO (version 11) (QSR International Pty Ltd, 2015), the first author undertook inductive, iterative and thematic coding (Boyatizis, 1998) and preliminary analysis, including cross-case analyses of individual participant group data. Analysis was discussed with the team. An Advisory Group, including former social workers, older people and family carers, met three times to discuss the study processes and findings, with input from care users and family carers also obtained. Case study site representatives were also consulted about findings. This article uses pseudonyms to respect confidentiality.

**Table 1.** Interview questions for local authority participants.

Local authority practitioners: Qualitative semi-structured interview guide
Do you signpost or refer to day centres for older people? Can you expand on the reasons for this?
Can you tell me about your opinions of day centres for older people and their purpose?
What, if anything, do you think day centres offer, or have the potential to offer, the health and social care market? Could you expand on this?
In your view, does [*name of day centre*] have a particular ‘unique selling point’? If yes, what would you say that is?
In your view, does [*name of day centre*] offer ‘added value’ somehow? If yes, what would you say that is?
Could you tell me about anything you think has influenced your views about day centres for older people?
Is there anything else you’d like to share with me about day centres for older people?
Local authority commissioners: Qualitative semi-structured interview guide
Do you/does [local authority] commission or fund any day centres for older people or any places at day centres? If yes, can you expand on the reasons for this? If no, did you ever commission day centres? Why did you stop?
Can you tell me about your opinions of the purpose and role of day centres for older people?
What, if anything, do you think day centres offer, or have the potential to offer, the health and social care market? If yes, could you expand on this?
In your view, does [*name of day centre*] have a particular ‘unique selling point’? If yes, what would you say that is?
In your view, does [*name of day centre*] offer ‘added value’ somehow? If yes, what would you say that is?
Thinking about data about day centre users, is there any data about users that may be helpful when making commissioning decisions and, in your view, day centres could collect?
Is there anything else you’d like to share with me about day centres for older people?

### Participant characteristics

This article draws on interviews with 23 attenders, 5 local authority participants responsible for commissioning services and market shaping, 8 frontline professionals (mainly social workers) who signposted or referred older people to day centres or other services, and 6 day centre managers or providers. Attenders’ characteristics, reasons for attendance and outcomes experienced are reported in detail elsewhere (Orellana et al., 2020a). To summarise, all attenders, whose average age was 83.3 years, reported health conditions or disabilities that impacted greatly on their life. Most had no partner. Three-quarters were women. Around two-thirds: lived alone; lived in rented homes; self-funded their attendance; were at greater risk of mental ill-health or isolation because of the limits of their social networks. Around half received means-tested benefits. Only one-quarter had continued education beyond secondary school. Apart from marital status and living arrangements, the profiles of attenders varied between centres reflecting local demographics.

Commissioners’ role titles included Coordinator, Commissioning Manager, Strategic Commissioner, Joint Commissioning Lead and Area Director. Three were responsible for commissioning older people’s services, two a broad range of services, and one a range of leisure, health and well-being services. Two commissioned services for different adult service user groups. Two had additional responsibilities outside social work services. They had been in their roles from one to ten years. Previous roles, all in local authorities, included managing a service operational team, commissioning (including services for people with learning disabilities), managing and developing leisure provision, and reviewing services.

Frontline professionals’ roles spanned local authority responsibilities, from initial access, screening and assessments to agreeing funding of care and support packages, reviewing care packages, some with older people and some with all adults. One had additional responsibilities for safeguarding (elder abuse) investigations and another for special projects. They had been in their roles from less than a year to seven years. Prior roles included running day centre classes for younger disabled people and people with learning disabilities, mental health outreach, social work, management and assistant director for adult services. One had been the assistant manager of a voluntary-run dementia day centre. Former roles outside social care were recruitment and charity project roles. As not all frontline participants were social workers, we refer to them as practitioners. The wider group of both commissioners and referrers is referred to as professionals.

## Findings

Findings are presented in four sections. Local authority professionals’ views of day centres’ relevance are summarised. Enactments of person-centredness and the lack thereof are exemplified, and, then, attenders’ perspectives of the positives and drawbacks of day centres’ congregate nature and the continuity of people they afford presented. Verbatim quotations from participants illustrate points being made, with underlined text indicating emphasis in the original. Pseudonyms are used for attenders and reference numbers for other participants.

### Day centres: Local authority professionals’ views

A higher proportion of practitioners than commissioners viewed day centres as relevant to contemporary policy imperatives and were also much more positive than commissioners about their part in the local care market, conceiving day centres as offering some choice and control. Practitioners spoke about the value of having a choice of centres or other services in the locality, choices of activities (which changed with changing clientele) and knowing an individual’s support needs. One commissioner perceived that monitoring health and well-being equated to a highly personalised service in which relationships had been built and saw this as potentially part of the role of day centres, thus linking them with Care Act imperatives of preventing or delaying deterioration. However, another commissioner was sceptical about the extent to which personalised services could be offered because attenders were ‘*not known to the system for very long. By the time you kind of develop a really good personalised offer, that person has deteriorated or gone to live in a care home or whatever*’ (Commissioner 5). She believed that delivering a range of appropriate activity choices was complicated by centres’ congregate nature. Her concerns were about lack of choice of, and flexibility between, days and times, inability to choose to do something different, limited meal options, not always having preferred staff, variations in forms of address, and lack of information given to families. In her view, an additional challenge to maintain a personalised approach was the need to remember to keep enquiring about attenders’ preferences.

### Enactment of person-centred approaches

#### The positives

Knowing people individually is central to personalised care and support that incorporates choice and control. Most practitioners reported trying to match a person to a centre which involved having visited it or knowing about its activities. Professionals made referrals or passed information to people about local centres which could include community centres with drop-in activities, structured (all-day) centres run by the local authority (in some of the areas) or voluntary sector, those for specific user groups (e.g. people from an ethnic group or with dementia) or centres where attenders had mixed needs. Mobility requirements needed to be considered. Community centres providing drop-in activities offered variety and tended to be cheaper but could be hard to physically access which meant that ‘*the structured day centres can be enticing because they offer transportation*’ (Practitioner 1). Another practitioner organised ‘test runs’ before potential attenders committed themselves to regular attendance, to enable informed decisions as reported by one attender:

*I came here on a trial basis for a week and at the end of the week, somebody came (…) and interviewed me (…) and she said, how would you like to start coming here three days a week? I went, “I'd love it''.* (Isobel)

A personalised approach seemed evident from the outset at most centres. One centre manager commented on how individual needs and preferences were built into support, such as how people were helped into and out of chairs or knowing their capabilities in order to encourage them to do what they can, commenting that maybe ‘*it’s probably never even looked at it like that*’ (Manager 1). One commissioner recognised another centre’s ‘*personalised, individual approach (…) in a very caring and supportive environment*’ (Commissioner 4) which they attributed to the provider’s set-up, reputation, ethos and personnel.

Pre-attendance assessments, which, in some centres became part of an individual’s care plan, revealed useful information that helped staff support new attenders. One attender had been accompanied to the centre from her home by a staff member who knew she was anxious and needed company on her first day. Being ‘buddied up’ or introduced to someone was common practice:

*She sat me next to [two attenders] who had been going, I gather, for about 12 years.* (Ruth)

Another had completed a form about herself and her interests on her first visit:

*I could write out what I was interested in (…). And I was very interested in Scrabble [board game] and they didn't do Scrabble. But she said, well you can see [staff], she deals with board games and things, so I did and she said “oh yes, you can have Scrabble. We've got a Scrabble board here,” and we started Scrabble*. (Francine)

Discovering interests continued after attendance became regular. One centre’s activity leader sought to discover people’s musical tastes and favourite songs; she played these weekly alongside discussion about them.

Another manager took a strengths-based approach and enquired if new attenders were interested in practical involvement (although the good mobility of the attender cited here was atypical):

*And she (…) gave me a chance to do certain jobs and afterwards I got to, like, (…) buy a bottle of milk or set tables up, that sort of thing.* (Lenny)

Another attender was supported by her key worker when official forms needed completion:

*So they read it for me (…) I can't read like that word – big words.* (Jane)

Despite difficulties with lateness of group transport, reported in the next section, experiences of transport suggest that, in the main, attenders’ needs were addressed individually; drivers knew names and requirements.

Day centre staff knew and understood attenders’ circumstances and reasons for attending; no attender inferred that those working in the centre did not know them quite or very well:

*They care, and they understand why I am here and what I am doing here and so on.* (Mariana; emphasis in the original).

‘Little touches’ further indicate how care and support can be delivered in a personalised way when a relationship exists between those providing and receiving support. For example, one staff member shared his family photos with Kaye in the knowledge that she loved children:

*He has two little boys and because I absolutely adore children (…) He always comes up and shows me photographs and he says, “I've got photographs of the boys.”* (Kaye)

Another attender commented that staff noticed if a person was ‘not themself’ and acknowledged this:

*If you are not well and they come and bring an extra tea in the afternoon.* (Mariana)

Regarding choice and control at centres, far from disempowering attenders and undermining their identity, attenders often exercised agency. They mainly reported feeling in control of what happened while at their centres. They chose where to sit, what hot drink or meal to have, and which activities to join in, reportedly without feeling pressured. It was common practice for attenders to be invited and encouraged to join in rather than assuming they would want to:

*Every time they always say, “You don't have to if you don't want to.”* (Isobel)

Sometimes, staff tried to find alternatives for those not joining in or made time to chat with them while activities were going on. Nellie enjoyed the organised group activities but preferred not to join some activities:

*I don’t join in a lot of the afternoon activities, because I do my knitting or crosswords (…) It’s just that I don’t play card games. I don’t play dominoes. So I’m quite happy to sit there in the afternoons and do my knitting.* (Nellie)

Francine chose to organise her own transport despite this not being the norm:

*They asked me about my transport and said “we can arrange transport.” I said “well, no”, I said, “because I like to go there at the time I want to go and come back at the same time.”* (Francine)

Such accounts and observations reveal that attenders felt they were not passively ‘done to’. Moreover, all answered without hesitation when asked if they would know what to do if unhappy with something at the centre and if they would be comfortable raising concerns. Responses suggested that most felt safe or assertive enough to raise problems and had confidence in the centre managers. Among those who did not feel comfortable doing so, reasons included feeling the manager was already overburdened, worry that a complaint might not be welcome and not wanting offending co-attenders to be embarrassed. Three reported having satisfactory experiences of raising concerns, one had kept concerns to herself and another was currently complaining about meals.

#### The negative

At macro level, certain choices were less feasible, mainly for systemic or operational reasons. Of the participants supported by social workers to attend their centres, half had not been offered choices of centre or other services, and one did not say whether options were offered; centres had either been recommended, were a second choice when the first was unavailable, or appeared on lists sent by the local authority after an enquiry. Limited operational days at voluntary sector centres restricted choice of attendance day(s). Choice of days or number of days was more relevant to the centres operating for five days; one-third of their attenders who raised matters concerning choice, had chosen on which days and the number of days to attend, and half as many had not been able to attend on additional days.

Individual needs and preferences were not always catered for at centres. There were not always parallel activity options, sometimes due to staff shortages, high support needs or venue logistics. Not all centres acted on their knowledge of individuals’ needs (e.g. sensory impairments), the reasons for which were not investigated; some attenders’ sensory impairments, therefore, affected their ability to enjoy or join in certain activities. One day centre had an audio system, but microphones were rarely used. One manager was considered to lack understanding of sight loss. A visually impaired attender did not chat on the minibus as she was unaware of who else was present, but familiarity of voices was helpful once at the centre:

*I sort of know what's going on, but a lot of things I don't understand because I can't see if they are doing games or questions and answers and things. It's a bit difficult.* (Ruby)

There was no choice of meals in one centre, but attenders could choose whether to have the meal provided; during fieldwork visits, one attender was offered an alternative from a local supermarket.

More broadly, some commissioners and practitioners felt that day centre use itself was not a choice but was needs-driven; need might have prompted action rather than an active choice taken to attend a day centre. They felt that those declining a day centre option were more likely to be mobile, or feel frail or unsociable, or had been put off by the term ‘day centre’ or had negative preconceptions of them. We have reported attenders’ reasons for attendance and their low awareness of day centres prior to attendance separately (Orellana et al., 2020a). Lenny (the atypically mobile attender attending a voluntary sector centre) acknowledged that day centres would not be everyone’s preference, but argued that whether to attend should be a choice:

*… to me it is the choice of the person (…) if they don’t want it, it’s up to them. When I found out* [about the centre]*, I was all for it. I took a chance. I enjoyed it. Now and then, I might wake up one morning, I don’t feel like coming in, I might have to, I don’t have to but I think I’d better ring (…), tell them I won’t be coming in.* (Lenny)

### Day centres’ congregate nature and the continuity they afford: Attenders’ perspectives

Attenders were asked about their two favourite and least favourite aspects of going to their day centre. All answered readily when asked about their favourite aspects and two-thirds were adamant they disliked nothing, even after prompting about specific things mentioned earlier in interviews or during fieldwork. The consideration given to this question before answering and the conviction of responses suggested attenders did not feel pressurised into false positivity. Most responses concerning the most and least favourite aspects were related to day centres’ group environment and the continuity they afford, as detailed in the next section. One, who said there was nothing she liked least, considered what she was unhappy with to be ‘*a little blip*’ which would pass (Ruby). Nevertheless, when asked for any suggestions they had to make their experience better only a small number of attenders said they would not change anything. Most were a mix of suggested service extensions (introduce toenail cutting, a sweet shop, raffles) or more of certain activities (board and card games, baking, parallel activities in small groups in different rooms), perhaps indicating very low dissatisfaction with current activities offered.

#### The positives

Positive views concerning the value of continuity and the setting’s congregate nature recurred across the various socio-demographic sub-groups, among self-funders and publicly funded attenders, and across day centres.

Continuity provided the opportunity to build trusting relationships with those providing the service. Care and support were provided in a kind, helpful, friendly and enabling way by staff and volunteers with whom attenders had built a relationship and whose character and behaviour attenders appreciated:

*They are so kind. They look after you.* (Kenneth)

Attenders reported that managers found time to speak to everyone, despite the demands of their job which they clearly cared about. They also liked that managers offered solutions to problems, were pleasantly straight-talking, always cheerful, lively, happy to be teased and gave the occasional kiss. Attenders considered frontline staff to be approachable, good listeners and understanding. Having smiling staff who were happy to have a laugh made for a better experience. Staff managed perceived rudeness and health crises well. Some attenders liked being assigned a key staff member. Staff’s (comparative) youth and liveliness helped one attender to feel younger and have fun. Volunteers looked after attenders well and were patient, despite always being busy. One attender was fond of a volunteer with a learning disability (intellectual impairment), appreciating her welcome and hugs; she also commented that this volunteer sometimes lost her temper. During fieldwork, personality clashes occurred between this and another volunteer and an attender, both with a learning disability; attenders appeared accustomed to this and may have felt a sense of reciprocity in that they felt able to provide their own support to this and other individuals.

A group environment enabled chatting during meals and getting a better perspective of one’s own situation by seeing people who seemed worse off:

*Just being able to chat to people when we go to dinner, we sit probably with somebody different and that's quite nice.* (Elizabeth)

*Well, coming here you meet, you meet other people, which some of them, you don't glad to see but they are in a worse condition than one might be.* (Denzel)

Having proper conversations and arguments, with people of their own age, and having a laugh and a joke were also among participants’ favourite things. The variety of characters made for different conversation topics. Attenders enjoyed received a smiling welcome and seeing familiar faces:

*Well it is the fact that everybody says, hello and you are welcomed; when you arrive, you see familiar faces even if you don’t talk to everybody*. (Ruth)

Continuity of company may have contributed to a sense of community. During fieldwork, the sense that centres were a community was evident in that there was manifest concern about absentees’ welfare. When one attender died, flowers were displayed and there was an air of sadness as staff informed attenders individually. When discussing social connections in interviews, many talked about former day centre attenders who had died or become ill and no longer attended.

#### The drawbacks

Their own and other attenders’ health conditions affected experiences. That fellow-attenders were ill, had moved to care homes or had died were upsetting but could be expected; occasionally, attenders experienced health crises at centres. One attender was irritated by the constant rocking of another attender with a learning disability. Seeing people with dementia, for a few, was sad.

Interaction with attenders with dementia commonly featured among dislikes expressed. For example, an aggressive reaction during a game had been taken personally, upsetting one attender who did not understand why some attenders disliked him, and another said ‘*Some are a little bit annoying (…) One time she started smacking people with her stick*’ (Elizabeth). Conversation was sometimes difficult:

*They talk silly things*. (Olive)

Lateness of transport also commonly featured. This mainly concerned people not being ready on time and a person with dementia who was, reportedly, confused about whether they had arrived at their final destination which caused difficulties at every pick-up; one attender had heard co-attenders complain ‘*that takes a half an hour off the time that we are there*’ (Kaye). It is worth noting that memory problems, such as those associated with dementia, played a role in transport timing. Those ‘not ready’ were likely to have forgotten what day it was:

*Quite a few of them aren't ready. That annoys me, because we are sitting on the bus and we've all got ready. The driver has to go out and bring them through and they haven't even got their coat on or their shoes on or whatever. That upsets me a bit. Then again, it's old people are like that, then so be it*. (Bob)

Some were more understanding than others, perhaps because they, themselves, found getting ready in the morning challenging. Some experienced fatigue during the day or afterwards, ascribing their lack of stamina to advanced age:

*I find, when you get to our age, after lunch you just sort of droop a little bit*. (Rosemary)

Although a few suggested that shortening the centre opening hours by half an hour or an hour would counteract this, most were happy with the day’s length and a few even felt it could be extended or operate for an additional day.

At one centre, physical disabilities also meant that staff needed to help people to the toilet which sometimes meant the main centre area seemed too quiet as staff were too busy to play games or put music on:

*When [staff] is there, she plays music. Sometimes some of them are too busy to get round to do it. (…) Sometimes one of them will come and sit down and play [dominoes] with [attender] and myself, or if [attender] isn’t there. By the time she sit down, she is called away to go to someone to go to the loo or someone to do something.* (Thomasina)

Finally, one attender considered the room layout to be constraining (compared with her previous much larger day centre with multiple rooms) and found the norms about where people sat for the various parts of the day restrictive.

## Discussion

This article contributes to the evidence by reporting factors related to day centres’ congregate nature and the enactment of person-centredness in settings that affect cognitively able older attenders’ access to these or their experiences. These findings demonstrate that receiving a service in a group setting does not fundamentally conflict with the concept of personalisation, which encompasses choice, control and person-centredness, and offers some advantages over individual support. Accessing day centres, however, may be compromised by systemic or operational factors.

Consideration of day centres in relation to the Care Act ambitions and its implementation is relevant to social work practice and may have international interest. Three points for discussion emerge from this empirical study: the operationalisation of choice and control in later life, factors relating to market shaping, and day centres’ congregate nature in the context of preventing social isolation and loneliness.

The first concerns the operationalisation of choice and control in accessing day centres and within the service itself. The Care Act placed a duty on local authorities to promote well-being, which is widely interpreted as improving outcomes by offering choice and control over care and support. Yet, tensions arise when implementation is within a context of funding reductions, when interpretations of what personalisation means differ, and when assumptions or experience of different client groups predominate over evidence. Personalisation covers care and support planning, individualisation of finances and support choices, yet for many older people exercising ‘consumer’ behaviour, there is reduced choice over what they can access due to day centre closures. In this present study there was the choice of accessing one centre at least; in other localities such a choice may no longer exist, leading to ‘enforced individualism’ (Roulstone & Morgan, 2009, p. 334).

Empowerment by staff is key to ‘good care’ (de São José et al., 2016; Fawcett, 2014) and while engagement in decision-making is central to service person-centredness (Wilberforce et al., 2017), individual older people have limited options, especially when unwell or disabled. In this present study, participating attenders were encouraged to exercise, or were supported to exercise, decisional autonomy (Söderberg et al., 2013). Most made active choices, both to attend their centre and within their centres on attendance days. Feeling in control is noted to be highly desirable and to significantly influence experiences (Glendinning, 2008) and quality of life (Boyle, 2004). Elsewhere, we have reported that the joint third highest domain of gain, when impact of attendance was measured using the Adult Social Care Outcomes Toolkit (ASCOT), was that of feeling in control of what attenders did at centres and when they did it (Orellana et al., 2020a). This contrasts starkly with reports of infantilisation and constraints to autonomy within some day centre settings (Liou & Jarrott, 2013, 2018; Salari, 2005). Additionally, this study’s attenders were assertively appreciative and knew how to deal with problems with the ultimate choice, in line with Sheikh et al.’s (2012) finding, to stop attending if unhappy with service quality. Attenders gave a strong impression that they were not coerced into attending nor were forced to stay. That said, there is an inherent risk of disregarding people’s individuality, or individual choices, when services are provided to groups, and service choices may not always be as person-centred as possible, particularly if funding constraints also restrict possibilities (see Needham et al., 2020). Organisational constraints inevitably affect service provision, sometimes limiting service user choice and autonomy (Hennessy, 1989). Ultimately, individuals have some choice to decide if and what to compromise in the pursuit of their service-related goals. Having the choice to attend their day centre, together with their experiences at them, may help explain, to an extent, why this group of attenders felt mainly happy with the levels of choice and control on offer. The limited contact of day centres with other social care provision may mean that opportunities to share good practice are under-developed.

Second, is the recurring theme of market shaping. This study provides further insights into professionals’ limited awareness of day centres. While social workers may acknowledge continuity of support and meeting of outcomes in the purchase of day services by an individual through a personal budget or Direct Payment, they may not perceive the additional opportunities provided by group settings. Among the most-liked aspects of day centres was the sense of continuity (value of personal biography), which others have observed to be one of the six ‘senses’ contributing to quality of care (Nolan et al., 2006). Continuity of staff and good relationships can hinder or help the achievement of what older people with high support needs want and value (Katz et al., 2011). Meaningful and trusting relationships are emphasised within personalisation (Needham & Glasby, 2015). As the wider literature notes, having built up a service user–staff relationship puts the former at ease and enables staff to learn about their preferences and dislikes ‘*which helps to embed personalised care*’ (ekosgen, 2013, p. iii) and to undertake the ‘small things’ that matter (Nolan et al., 2006). It is well established that relationship-centred care is grounded on interactions between providers and service users (Tresolini & Pew-Fetzer Task Force, 1994), and older people seem to place value not only on relationships with care workers but also staff’s personal characteristics (Manthorpe et al., 2017); kindness, warmth, compassion, friendliness (especially for those who were isolated or lived alone) and a personal approach were of particular note in our study. Findings offer further insight into contributors to the outcome of gaining a ‘personal sense of significance’ (dignity) which we have reported elsewhere (Orellana et al., 2020a), something that may be more achievable in group settings, where significance is acknowledged and affirmed by multiple people, than in individualised ‘out and about’ services, or services delivered individually in the home. A literature review and concept mapping undertaken to develop a new care experience measure, the person-centred community care inventory (PERCCI), confirmed that understanding the person and promoting the care relationship are central to service person-centredness; within these categories, continuity of care and personal qualities (of staff) and relationships contribute to experience (Wilberforce et al., 2017; Wilberforce, Batten, et al., 2018; Wilberforce, Challis, et al., 2018).

Thus, while their congregate nature is a feature for which day centres are criticised, when older people are asked it is core to many of attenders’ favourite centre-related experiences and outcomes (see Orellana et al., 2020a). It appears to counteract, to a degree, the drawbacks of ageing in place in isolation, with its risks of loneliness, and is, therefore, a strength.

The positives appeared to outweigh attenders’ least favourite aspects of centres. There were some drawbacks to a group environment, yet people still attended regularly despite the unwelcome impact to some of cognitively impaired co-attenders and the degree of environmental inflexibility complained about by a minority. Their dislike of transport delays may also indicate how strongly transport contributed to experiences. Personalisation of transport may be a focus that could improve centres’ effectiveness.

## Implications for social work practice and policy

Benefits of togetherness in social care are lost if group environments are not financially and conceptually supported as part of market shaping. This is important at a time when the negative health impacts of social isolation and loneliness are being emphasised in England (HM Goverment, 2018). Withdrawing financial support for day centres and enforcing service individualisation appear to have created new inequalities as local authorities have found difficulty in sustaining investment in existing services, still less prevention, in their highly constrained resource environment.

Creativity in care and support planning, and the maximisation of local assets, through the use of community resources are encouraged by the Care Act (HM Government, 2014). However, even with individual support, getting ‘out and about’ would be challenging given the substantial levels of disability experienced by many participants in this present study (e.g. needing help to stand up and with personal care).

Social workers who commission or refer to day centres and those taking management decisions about such referrals may wish to visit day centres and talk to attenders to improve their understanding of them. Use of the PERCCI tool or of ASCOT in day centres could be one way to furthe r evidence levels of person-centredness.

## Limitations of the study

Centre diversity and the identification of common themes across centres help compensate for the study’s limitations which relate to the small number of participating centres in one English region and small sample sizes. Frailer and less cognitively able attenders may have been under-represented, although, in conversation during visits, many non-participants expressed similar views and experiences as participants. A risk of bias is that poor-quality day centres may not have agreed to participate. The study’s strengths lie in its focus on generalist day centres and its in-depth nature. Rigour was maximised by lay scrutiny of interview questions. Care was taken to overcome risks of people fearing they might lose their day centre place if comments were negative, and the possible tendency to answer positively to all questions or wish to please the researcher was moderated by development of familiarity and a rapport with the researcher during the visit period and by most interviews taking place away from day centres. Findings’ transparency and trustworthiness were reinforced by feedback from the study Advisory Group and case study site representatives.

## Conclusions

This article challenges the view that day centres’ group nature means they are outdated and irrelevant. Of central importance to day centre experiences are some of the stereotypes driving their poor image, closure and decommissioning. For some older people, there seems value in the day centres’ collective provision which suggests this not in direct conflict with the principles of the Care Act, subject to service quality. Government priorities to reduce social isolation may be supported by day centres’ congregate nature.

The continuity day centres afford underpins person-centredness within them. There is evidence that attenders are treated as individuals, achievement of which requires constant awareness of practices. However, since process outcomes depend on service quality, service person-centredness, choice and control exerted and the benefits of day centres’ congregate environment may be less evident in poorer quality services or in services with high proportions of distressed or cognitively impaired people.

In thinking about day centres, the focus must shift from service format (congregate) to the ‘software’ (culture) of the service. Further research might explore what creates the culture within a centre and links between culture and outcomes. This may address, for example, what a manager’s role involves, what culture prevails and the role it plays, what role the building/environment plays and whether this transcends a manager’s influence. Findings may inform the development of a model of day centre culture, as there is for care homes (see www.myhomelife.org.uk).

An asset-based community development approach could focus on the benefits of a group setting for some people, such as day centre staff’s knowledge of individual attenders and centres as an opportunity for building social connections and a sense of mutual support, particularly for people with limited social networks. Social workers’ development of strengths-based practice may include building on such community capacity as recovery from the COVID-19 pandemic begins.
